# Low-moderate urine arsenic and biomarkers of thrombosis and inflammation in the Strong Heart Study

**DOI:** 10.1371/journal.pone.0182435

**Published:** 2017-08-03

**Authors:** Katherine A. Moon, Ana Navas-Acien, Maria Grau-Pérez, Kevin A. Francesconi, Walter Goessler, Eliseo Guallar, Jason G. Umans, Lyle G. Best, Jonathan D. Newman

**Affiliations:** 1 Department of Epidemiology, Johns Hopkins Bloomberg School of Public Health, Baltimore, MD, United States of America; 2 Department of Environmental Health Sciences, Johns Hopkins Bloomberg School of Public Health, Baltimore, MD, United States of America; 3 Institute of Chemistry–Analytical Chemistry, University of Graz, Graz, Austria; 4 MedStar Health Research Institute, Hyattsville, MD, United States of America; 5 Missouri Breaks Industries Research, Timber Lake, SD, United States of America; 6 New York University School of Medicine, New York, NY, United States of America; BRAC, BANGLADESH

## Abstract

The underlying pathology of arsenic-related cardiovascular disease (CVD) is unknown. Few studies have evaluated pathways through thrombosis and inflammation for arsenic-related CVD, especially at low-moderate arsenic exposure levels (<100 μg/L in drinking water). We evaluated the association of chronic low-moderate arsenic exposure, measured as the sum of inorganic and methylated arsenic species in urine (ΣAs), with plasma biomarkers of thrombosis and inflammation in American Indian adults (45–74 years) in the Strong Heart Study. We evaluated the cross-sectional and longitudinal associations between baseline ΣAs with fibrinogen at three visits (baseline, 1989–91; Visit 2, 1993–95, Visit 3, 1998–99) using mixed models and the associations between baseline ΣAs and Visit 2 plasminogen activator inhibitor-1 (PAI-1) and high sensitivity C-reactive protein (hsCRP) using linear regression. Median (interquartile range) concentrations of baseline ΣAs and fibrinogen, and Visit 2 hsCRP and PAI-1 were 8.4 (5.1, 14.3) μg/g creatinine, 346 (304, 393) mg/dL, 44 (30, 67) mg/L, and 3.8 (2.0, 7.0) ng/mL, respectively. Comparing the difference between the 75^th^ and the 25^th^ percentile of ΣAs (14.3 vs. 5.1 μg/g creatinine), ΣAs was positively associated with baseline fibrinogen among those with diabetes (adjusted geometric mean ratio (GMR): 1.05, 95% CI: 1.02, 1.07) not associated among those without diabetes (GMR: 1.01, 95% CI: 0.99, 1.02) (p-interaction for diabetes = 0.014), inversely associated with PAI-1 (GMR: 0.94, 95% CI: 0.90, 0.99), and not associated with hsCRP (GMR: 1.00, 95% CI: 0.93, 1.08). We found no evidence for an association between baseline ΣAs and annual change in fibrinogen over follow-up (p-interaction = 0.28 and 0.12 for diabetes and non-diabetes, respectively). Low-moderate arsenic exposure was positively associated with baseline fibrinogen in participants with diabetes and unexpectedly inversely associated with PAI-1. Further research should evaluate the role of prothrombotic factors in arsenic-related cardiovascular disease.

## Introduction

Almost five million people in the United States (US) drink water from public and private wells with arsenic concentrations above US Environmental Protection Agency (EPA) standard of 10 μg/L [1–4], and millions more are exposed below this level. Drinking water and food are important sources of inorganic arsenic exposure in populations with low levels of arsenic in drinking water [4–8]. Taken together, epidemiological studies of populations with high (≥100 μg/L) [9–11], and low-moderate (<100 μg/L) levels of arsenic in drinking water [12–14] support a causal link between chronic arsenic exposure and cardiovascular disease (CVD), particularly coronary heart disease (CHD) [1]. The underlying etiology of arsenic-related CVD, however, has not been established [15, 16].

Inflammation and thrombosis via increased coagulation and decreased fibrinolysis are hallmarks of the initiation and progression of atherosclerosis [17, 18]. Higher levels of fibrinogen, a major coagulation factor related to inflammation and vascular thrombosis, and CRP, a biomarker of systemic inflammation, have been consistently associated with incident CHD and stroke in large individual participant meta-analyses from prospective cohort studies [19, 20]. Plasminogen activator inhibitor-1 (PAI-1), a major inhibitor of the fibrinolytic system, has been associated with incident CHD in some studies [21], but not in others [22].

Diabetes is a strong risk factor for CVD [23, 24], but differences in traditional risk factors for CVD (e.g., dyslipidemia and hypertension) do not entirely explain the association between diabetes and incident CVD [25]. Concentrations of plasma fibrinogen, PAI-1, and CRP are higher in individuals with diabetes [26], and many of the shared pathological changes associated with initiation and progression of atherosclerosis and diabetes are linked to insulin resistance [27].

Chronic arsenic exposure has been associated with higher plasma PAI-1 [28, 29] and CRP [30, 31] concentrations in a few clinical or cross-sectional epidemiologic studies of populations exposed to high levels of arsenic in drinking water (>100 μg/L). A small clinical study found higher levels of fibrinogen in subjects with Blackfoot disease, a peripheral vascular disease related to endemic high arsenic exposure in Taiwan, compared to controls [32]. No previous general population epidemiologic study, to our knowledge, has examined the association between chronic arsenic exposure and plasma fibrinogen. In *in vitro* and animal studies, exposure to arsenic increased PAI-1 concentrations and activity [33], CRP concentrations [34], and platelet aggregation [35].

We previously reported an association between chronic arsenic exposure and incident fatal and non-fatal CVD in the Strong Heart Study (SHS) [12], a prospective, population-based cohort of American Indians exposed to low-moderate levels of arsenic in drinking water. The objective of the current study was to examine the association between chronic arsenic exposure and three biomarkers of thrombotic risk and/or vascular inflammation, plasma fibrinogen, PAI-1, and CRP, in the SHS. Post-hoc subgroup analyses from previous epidemiologic studies in the SHS demonstrated that associations between urine arsenic and incident CVD and CHD were stronger among participants with diabetes [12], and CRP was associated with incident CVD only in those without diabetes [36]. Therefore, we hypothesized that associations between urinary arsenic and thrombotic/vascular inflammatory markers might differ by diabetes status.

## Methods

### Study population

A population-based longitudinal study of CVD and its risk factors, the SHS main cohort examined 4549 men and women age 45 to 74 years in 13 American Indian communities in Arizona, Oklahoma, and North and South Dakota in 1989–91 (Visit 1), with two follow-up exams in 1993–95 and 1998–99 (Visit 2 and Visit 3). Details of the study design have been decribed previously [37, 38]. At baseline, the participation rate in the main cohort was 62% [39], and 88% and 89% of surviving participants were examined at Visit 2 and Visit 3, respectively [40]. Compared to non-participants at baseline, participants were similar in age, body mass index, and the prevalence of diabetes, but were more likely to be female and to have hypertension [39]. The Indian Health Service institutional review board, institutional review boards of participating institutions, and participating tribes approved the study protocol. All participants provided informed consent.

Urine arsenic was measured at baseline among 3974 participants with available stored urine samples. In 2016, one community withdrew their consent to participate in further research, and we have excluded their data (N = 1033) from this analysis. Plasma fibrinogen was measured at all three visits, while PAI-1 and CRP were only measured at Visit 2. First, we examined the cross-sectional and longitudinal associations between baseline arsenic with repeated measures of fibrinogen at Visits 1, 2, and 3 in 2700 participants without prevalent CVD, complete baseline measurements of urine arsenic and creatinine, plasma fibrinogen, and key baseline CVD risk factors. Second, we examined the association between baseline arsenic and plasma PAI-1 and CRP at Visit 2 among 1984 participants without prevalent CVD at or before Visit 2, complete baseline measurements of urine arsenic, creatinine, key baseline CVD risk factors, and plasma PAI-1 and CRP at Visit 2. Prevalent CVD was defined as a history of definite or possible CHD, definite or possible myocardial infarction, definite or possible stroke, transient ischemic attack, and other CVD events [38]. We present the full inclusion and exclusion criteria for the analyses in the SHS main cohort in **S1 Fig**. Compared to the overall cohort (N = 3265, excluding participants who withdrew consent and those with prevalent CVD), participants in this analysis at baseline (N = 2700) were generally similar across socio-demographic and cardiovascular risk factors.

In a secondary analysis, we also examined the cross-sectional association between arsenic and plasma fibrinogen, PAI-1, and CRP in a subset of participants without diabetes at baseline in the Strong Heart Family Study (SHFS), an ancillary study of the SHS main cohort. The SHFS is a family-based longitundinal study of participants from the main SHS cohort and their family members age 14 or older [41]. Participants from large, multigenerational families were examined either during a pilot study during Visit 3 of the SHS main cohort (1998–99) or at the SHFS baseline Visit 4 (2001–03). Fibrinogen and PAI-1 were measured at both Visit 3 pilot or Visit 4, while CRP was measured only at Visit 4. Of 2919 SHFS participants who have given permission to conduct further research, urine arsenic was measured in 1948 (67%) participants who were without diabetes at baseline (Visit 3 pilot or Visit 4), were examined at a follow up visit in 2006–2009, and had sufficient stored urine for a study of environmental and genetic risk factors of incident diabetes (prevalent cases of diabetes were excluded from metals analysis). We further excluded participants missing either plasma biomarkers or key covariates for a final sample size of 1901 for fibrinogen and PAI-1 and 1791 for CRP (**S2 Fig**).

### Plasma fibrinogen, PAI-1, and CRP

Fibrinogen was measured in plasma by a modification of the von Clauss method [42] and plasma PAI-1 antigen and high-sensitivity CRP were measured by an enzyme-linked immunoabsorbent assay [43, 44]. Detailed laboratory methods for the SHS have been reported previously [38, 41]. The inter-assay coefficient of variation for fibrinogen, PAI-1, and CRP were <8%, 8%, and <5%, respectively [45].

### Urine arsenic (inorganic arsenic and methylated metabolites)

The analytical methods and associated quality control criteria for urine arsenic measurements in the SHS main cohort and family study have been described previously [46]. Arsenic species were measured in stored urine samples collected at the baseline clinical exam in 2009 and 2012 at the Trace Element Laboratory of the University of Graz (Graz, Austria) using high performance liquid chromatography (HPLC; Agilent 1100, Agilent Technologies) coupled to inductively coupled plasma mass spectrometry (ICPMS; Agilent 7700xICPMS, Agilent Technologies).

In the SHS main cohort, the inter-assay coefficients of variation (CV) for inorganic arsenic, MMA, DMA, and arsenobetaine for an in-house reference urine were 6.0%, 6.5%, 5.9%, and 6.5%, respectively [46]. In the family study cohort, the inter-batch variability was checked by replicate measurements of the arsenic compounds in three certified reference materials (NIST 2669 I, NIST 2669 II and NIES 18). The CV ranged from 5.4 to 14.4% for inorganic arsenic, 4.8 to 8.6 for MMA, and 5.5 to 8.1% for DMA (N = 46). The LOD for inorganic arsenic (arsenite and arsenate), MMA, DMA, and arsenobetaine plus other arsenic cations was 0.1 μg/L. For samples with arsenic species below the LOD (5.2% for inorganic arsenic, 0.8% for MMA, 0.03% for DMA, and 2.1% for arsenobetaine plus other cations), concentrations were imputed as the corresponding LOD divided by the square root of two [47, 48].

We used the sum of inorganic (arsenite and arsenate) and methylated (MMA and DMA) arsenic species (ΣAs) as a proxy for inorganic arsenic exposure. Urine arsenic concentrations were divided by urine creatinine concentrations to account for variability in dilution of the random urine samples, and expressed in μg/g creatinine. We conducted two sensitivity analyses to examine potential bias from dividing urine arsenic concentrations by urine creatinine, which is associated with muscle mass and nutritional status [49]. First, we conducted the analyses using urine arsenic concentrations without dividing by creatinine, and adjusted for log-transformed urine creatinine concentrations in regression models. Second, in the subset of participants without diabetes or albuminuria, we conducted the analyses using baseline arsenic concentrations divided by specific gravity [49]. These sensitivity analysis results were consistent with the main analysis.

### Other variables

For both the SHS main cohort and SHS family study, each study visit consisted of a personal interview and clinical examination, with blood and urine samples collected in the morning after at least a 12-hour overnight fast. A full description of the standardized methods and protocols have been reported previously for the main cohort [38] and family study [41].

Definitions of sociodemographic and CVD risk factors were largely standardized across the SHS main cohort and family study. We defined hypertension as systolic blood pressure of 140 mm Hg or greater, diastolic blood pressure of 90 mm Hg or greater, or antihypertensive medication use [50]. In the main cohort, low-density lipoprotein (LDL) cholesterol levels were calculated using the Friedewald equation [51] and missing values were replaced with measured LDL cholesterol using the beta quantification procedure [38]. We defined albuminuria as a urine albumin to creatinine ratio of 30 mg/g or greater [52]. We estimated glomerular filtration rate [eGFR) from recalibrated plasma creatinine measurements [53], using the Chronic Kidney Disease Epidemiology Collaboration equation [54]. In the main cohort, we defined diabetes as a fasting glucose level 7.0 mmol/L or greater (126 mg/dL), two-hour post-load plasma glucose level 11.1 mmol/L or greater (200 mg/dL), hemoglobin A1c level 6.5% or greater, or self-reported use of insulin or an oral hypoglycemic agent [55]. In the family study, the analysis was conducted only among persons without diabetes, defined by reported use of insulin or oral diabetes medication or a fasting plasma glucose concentration ≥126 mg/dL (7.0 mmol/L) [56].

### Statistical analysis

Fibrinogen, PAI-1, and CRP concentrations were log-transformed concentrations in regression models to improve normality. All analyses were a priori stratified by diabetes status because we previously found a stronger association between arsenic exposure and incident CVD [12] in individuals with diabetes in the SHS, and CRP was only associated with CVD among individuals without diabetes in the SHS [36].

For plasma fibrinogen, we used a linear mixed effects model to evaluate the cross-sectional and longitudinal associations between baseline urine arsenic and repeated measures of fibrinogen at baseline, Visit 2, and Visit 3 in the SHS main cohort stratified by diabetes at baseline. Modeling repeated measures of fibrinogen allows participants without a follow-up visit to be included, and improves the precision of the cross-sectional estimates [57]. We included fixed effects for baseline arsenic, years from baseline visit, an interaction between baseline arsenic and years from baseline, and potential confounders measured at baseline. The best-fitting model, determined by the likelihood ratio test and Akaike Information Criterion (AIC), included random subject-specific intercepts and slopes and allowed for correlation between the random intercept and slope. We found that there was no significant interaction between arsenic (log-transformed continuous and quartiles) and time, and subsequent models included only the main effects of arsenic and time. We used linear regression to examine the association between baseline urine arsenic and concentrations of PAI-1 and CRP at Visit 2 stratified by diabetes status (diabetes at either baseline or Visit 2).

For all models, we expressed the adjusted association between urine arsenic concentrations and each plasma biomarker as the geometric mean ratio (GMR) and 95% confidence interval of the plasma biomarker concentrations for a specified difference in urine arsenic concentrations. Urine arsenic concentrations were modeled as quartiles, log-transformed continuous concentrations, and as restricted quadratic splines of log-transformed concentrations with knots at the 10^th^, 50^th^, and 90^th^ percentiles. Quartiles of urine arsenic (μg/g creatinine) were created separately for the model of baseline fibrinogen (N = 2700) and models of Visit 2 PAI-1 and CRP (N = 1984). We controlled for potential confounding in sequential models. Model 1 was adjusted for age, sex, and education (no, some, or finished high school), smoking (never, former, current), and alcohol drinking (never, former, current), BMI (kg/m^2^), LDL cholesterol (mg/dL), hypertension (yes/no), eGFR (mL/min/1.73 m^2^), and diabetes status (overall models only). Model 2, the primary model, was additionally adjusted for study center (Arizona, Oklahoma, North and South Dakota). Model 3 additionally adjusted for albuminuria (ACR <30 mg/g, >30 to <300 mg/g, and ≥300 mg/g) and hemoglobin A1c (%). In the SHS, arsenic was cross-sectionally associated with albuminuria [58], and with hemoglobin A1c among those with diabetes [59]. Albuminuria and hemoglobin A1c may act as confounders or mediators of the association between arsenic and plasma biomarkers of thrombotic risk and vascular inflammation; therefore, we adjusted for these variables only in sensitivity analyses. Model 4 adjusted for the same covariates in Model 2 without diabetes status. While some evidence suggests that hypertension could also be a mediator of the association between arsenic and cardiovascular disease [60], urine arsenic is not associated with blood pressure or hypertension in this cohort. Additional adjustment for menopausal status among women did not materially change the associations.

For the plasma biomarkers that we observed an association with urine arsenic concentrations, we also examined whether there was an association with arsenic metabolism (urine %iAs, %MMA, and %DMA) in separate models. These models adjusted for covariates in Model 2 (fully-adjusted) and urine arsenic exposure (log-transformed). We conducted subgroup analyses to evaluate effect modification by selected participant characteristics in fully adjusted models by including interaction terms between log-transformed urine arsenic and an indicator variables for each categorical participant characteristic. Except for diabetes status, all subgroup analyses were exploratory without prior hypotheses. We found similar results when excluding participants with CRP concentrations above 10 mg/L, which may reflect acute inflammation [61].

In secondary analyses, we estimated the cross-sectional association between baseline (Visit 3 pilot/Visit 4) urine arsenic and plasma fibrinogen, PAI-1, and CRP in SHFS participants without diabetes (metals were only measured in participants without prevalent diabetes by design) using linear mixed models. We included a random effect for family to account for possible correlation within families. Consistent with the main analysis, we expressed adjusted associations as GMR and modeled urine arsenic exposure as quartiles, log-transformed concentrations, and as restricted quadratic splines of log-transformed concentrations with knots at the 10^th^, 50^th^, and 90^th^ percentiles. We adjusted for the same baseline covariates as in SHS main cohort analyses, with the exception that we adjusted for fasting plasma glucose instead of hemoglobin A1c because hemoglobin A1c was not available in most SHFS participants.

Statistical analyses were performed with Stata Version 12.1 (StataCorp, College Station, TX, USA) and R Version 3.2.2 (R Foundation for Statistical Computing, www.r-project.org, Vienna, Austria). All statistical tests were two-sided and p-values less than 0.05 were considered statistically significant.

## Results

### Baseline characteristics of SHS main cohort participants

At the SHS main cohort baseline visit (N = 2700), the median (IQR) age was 55 (49, 62) years, 59% of participants were female, 42% of participants had diabetes, and the median (IQR) concentration of urine arsenic was 8.4 (5.1, 14.3) μg/g creatinine (**Table 1**). At baseline, the median (IQR) of fibrinogen was 286 (244, 336) mg/dL. At Visit 2, median (IQR) concentrations of fibrinogen, PAI-1 and CRP were 346 (304, 393) mg/dL, 44 (30, 67) ng/mL, and 3.8 (2.0, 7.0) mg/L, respectively. Urine arsenic concentrations were highest in Arizona (median 17.2 μg/g creatinine), and lowest in Oklahoma (median 5.6 μg/g creatinine). Participants with higher urine arsenic had lower education, a higher prevalence of albuminuria, and were more likely to have diabetes, to drink alcohol, and have higher hemoglobin A1c. Selected participant characteristics by diabetes status and quartiles of urine arsenic are presented in **S1 Table**.

**Table 1:** Selected characteristics of Strong Heart Study main cohort participants at baseline (Visit 1) by quartiles of baseline urine arsenic (ΣAs, μg/g creatinine).

		Quartiles of Urine Arsenic (ΣAs, μg/g creatinine)				
	Overall N = 2700	Q1 N = 676	Q2 N = 675	Q3 N = 677	Q4 N = 672	
Mean (SD)	11.6 (10.9)	3.8 (0.9)	6.7 (0.9)	11.1 (1.7)	24.9 (14.4)	
Median (IQR)	8.4 (5.1, 14.3)	3.8 (3.1, 4.5)	6.6 (5.9, 7.4)	10.9 (9.6, 12.5)	20.4 (16.6, 27.1)	**p-**
Range	1.6, 179.9	1.6, 5.1	5.2, 8.4	8.4, 14.3	14.3, 179.9	value *
Age, years	55 (49, 62)	55 (49, 62)	55 (49, 62)	54 (49, 61)	55 (49, 63)	0.70
Female, %	1600 (59%)	332 (49%)	413 (61%)	414 (61%)	441 (66%)	**<0.001**
Finished high school, %	1602 (59%)	469 (69%)	438 (65%)	379 (56%)	316 (47%)	**<0.001**
Current smoker, %	1020 (38%)	233 (34%)	248 (37%)	274 (40%)	265 (39%)	0.10
Current drinker, %	1157 (43%)	237 (35%)	262 (39%)	333 (49%)	325 (48%)	**<0.001**
BMI, kg/m^2^	30 (26, 34)	30 (27, 34)	30 (26, 34)	30 (26, 34)	29 (26, 33)	**0.03**
Hypertension, %	960 (36%)	251 (37%)	248 (37%)	230 (34%)	231 (34%)	0.51
Diabetes, %	1145 (42%)	240 (36%)	266 (39%)	279 (41%)	360 (54%)	**<0.001**
Hemoglobin A1c, %	5.4 (4.9, 6.8)	5.3 (4.9, 6.0)	5.4 (4.9, 6.3)	5.4 (4.9, 6.7)	5.6 (5.0, 9.0)	**<0.001**
LDL cholesterol, mg/dL	118 (97, 140)	121 (100, 144)	121 (100, 142)	119 (97, 138)	113 (92, 137)	**<0.001**
eGFR, mL/min/1.73 m^2^	100 (91, 107)	98 (88, 106)	100 (91, 107)	101 (91, 107)	103 (94, 110)	**<0.001**
Albuminuria (ACR ≥ 30 mg/g)	629 (23%)	102 (15%)	131 (19%)	143 (21%)	253 (38%)	**<0.001**
Post-menopause, % of women	1212 (76%)	251 (76%)	310 (75%)	311 (75%)	340 (77%)	0.89
Fibrinogen, mg/dL (Visit 1)	286 (244, 336)	278 (242, 320)	282 (242, 326)	282 (242, 332)	293 (250, 354)	**<0.001**
Fibrinogen, mg/dL (Visit 2)	346 (304, 393)	334 (291, 381)	347 (304, 393)	339 (299, 388)	362 (320, 411)	**<0.001**
Fibrinogen, mg/dL (Visit 3)	363 (311, 428)	352 (298, 416)	364 (310, 429)	360 (313, 420)	381 (327, 454)	**<0.001**
PAI-1, ng/mL (Visit 2)	44 (30, 67)	49 (33, 72)	48 (32, 70)	40 (28, 60)	39 (28, 64)	**<0.001**
CRP, mg/L (Visit 2)	3.8 (2.0, 7.0)	3.4 (1.8, 6.1)	4.0 (2.0, 6.9)	4.0 (2.1, 7.6)	4.1 (2.0, 7.3)	**0.02**

SD, standard deviation; IQR, interquartile range; ACR, Albumin to creatinine ratio in urine; LDL, Low density lipoprotein; eGFR, estimated glomerular function; BMI, body mass index; PAI-1, Plasminogen activator inhibitor-1; hsCRP, high-sensitivity C-reactive protein.

Values are median (interquartile range) for continuous variables and number of participants (percentage) for categorical variables.

* P-values from a nonparametric Kruskal-Wallis test of difference in distribution (continuous variables) or Pearson’s chi-square test of independence (categorical variables).

We present median concentrations of plasma fibrinogen, PAI-1, and CRP at Visit 2 by diabetes status (at either baseline or Visit 2) and selected participant characteristics in **Table 2**. Associations between participant characteristics and baseline plasma fibrinogen were consistent with fibrinogen at Visit 2. In general, participants with higher plasma fibrinogen, PAI-1, and CRP were more likely to be female and had higher BMI. Higher fibrinogen concentrations were also associated with older age, higher hemoglobin A1c, higher prevalence of albuminuria, and lower education. In both participants with and without diabetes, however, plasma PAI-1 concentrations were lower in older participants and lower in those with reduced kidney function (eGFR <60 mL/min/1.73 m^2^). In participants with diabetes, PAI-1 concentrations were also inversely associated with SBP, hemoglobin A1c, and education (**Table 2**).

**Table 2:** Concentrations of plasma fibrinogen, PAI-1, and CRP at Visit 2 by participant characteristics and diabetes status at baseline in Strong Heart Study main cohort participants.

	Without Diabetes (N = 899)								With Diabetes (N = 1085)							
		Fibrinogen (mg/dL)		PAI-1 (ng/mL)			CRP (mg/L)			Fibrinogen (mg/dL)		PAI-1 (ng/mL)			CRP (mg/L)	
	%	Median	p-value	Median		p-value	Median	p-value	%	Median	p-value	Median	p-value		Median	p-value
Age, years *																
≤ 55	57%	325	**<0.001**	44		**0.003**	3.2	0.95	51%	350.5	**0.04**	50	**<0.001**		4.7	**0.002**
> 55	43%	341.5		38			3.2		49%	361		44			3.8	
Sex																
Male	43%	327	**0.002**	39		0.06	2.6	**<0.001**	34%	339.5	**<0.001**	45	0.05		3.1	**<0.001**
Female	57%	337		42			3.6		66%	364		48			5	
Education																
< HS	33%	341	**0.002**	44		0.15	3.4	0.14	43%	367	**<0.001**	43	**<0.001**		4.3	0.66
≥ HS	67%	330		39			3		57%	349		50			4.2	
Smoking																
Never/former	58%	329	**0.001**	40		0.16	2.9	**0.02**	69%	357.5	0.78	46	0.26		4.3	0.74
Current	42%	340		42			3.6		31%	355		47			4.2	
Drinking																
Never/former	51%	336	**0.04**	42		0.42	3	0.42	64%	361	**0.003**	46	0.45		4.4	**0.02**
Current	49%	330		40			3.3		36%	346		47			4	
BMI, kg/m^2^																
< 30	66%	330	**0.01**	37		**<0.001**	2.8	**<0.001**	40%	346	**0.001**	42	**<0.001**		3.7	**<0.001**
≥ 30	34%	338		49			3.7		60%	364		51			4.8	
Hypertension																
No	74%	332	**0.03**	39		**0.01**	3.1	0.07	59%	350	0.05	47	0.44		4.4	0.29
Yes	26%	343		44			3.5		41%	363		46			4	
Hemoglobin A1c, % †																
<5.7	83%	332	**0.03**	39	**0.02**		3	**0.02**	33%	331	**<0.001**	48	0.11	4.1		0.06
≥5.7	11%	338		47			4.1		60%	365		46		4.4		
LDL cholesterol, mg/dL																
<100	22%	333.5	0.90	44	0.26		3.1	0.88	30%	361	0.70	44	0.12		4.5	0.22
≥100	78%	334		39			3.2		70%	354		47			4.2	
eGFR, mL/min/1.73 m^2^																
≥60	99%	334	0.62	41	**0.03**		3.1	0.57	97%	354.5	**<0.001**	46	0.05		4.2	0.24
<60	1%	338		29			3.8		3%	407		40			5.6	
Albuminuria																
No	93%	332	**0.002**	40	0.16		3.1	**0.02**	68%	343	**<0.001**	48	0.07		4.1	0.11
Yes	7%	356		46			4.7		32%	388		45			4.8	
Menopause ‡																
No	16%	326	**0.003**	44	0.41		3.6	0.94	16%	356	**0.02**	53.5	0.05		6	**0.008**
Yes	41%	342		41.5			3.6		51%	365		47			4.8	

HS, High School; eGFR, estimated glomerular function; LDL, low-density lipoprotein; SBP, systolic blood pressure; BMI, Body mass index p-values are from a non-parametric Kruskall-Wallis test of equality of populations.

* Dichotomized at the overall median.

† Hemoglobin A1c was measured in 93% of participants.

‡ Among women only.

At Visit 2, CRP concentrations were moderately correlated with fibrinogen (Spearman ρ = 0.46, p<0.001) and PAI-1 (ρ = 0.26, p<0.001), while fibrinogen and PAI-1 showed little correlation (ρ = 0.06, p = 0.002). Correlations were similar by diabetes status.

### Cross-sectional and longitudinal associations between baseline urine arsenic and fibrinogen in the SHS main cohort

Of the 2700 participants with complete data at the SHS main cohort baseline, 78% and 69% had fibrinogen measurements at Visit 2 and Visit 3, respectively. Participants had a mean (standard deviation) of 6.2 (3.0) years of follow-up. As expected, plasma fibrinogen levels increased over time, with each year after baseline associated with a 4% increase (GMR: 1.04, 95% CI: 1.03, 1.04) in fully adjusted models (Model 2). In fully adjusted models (Model 2), we found no evidence of an interaction between baseline urine arsenic concentrations and the annual change in plasma fibrinogen over follow-up (log-transformed baseline arsenic, p-interaction = 0.28 and 0.12 for diabetes and non-diabetes, respectively).

**Fig 1:**
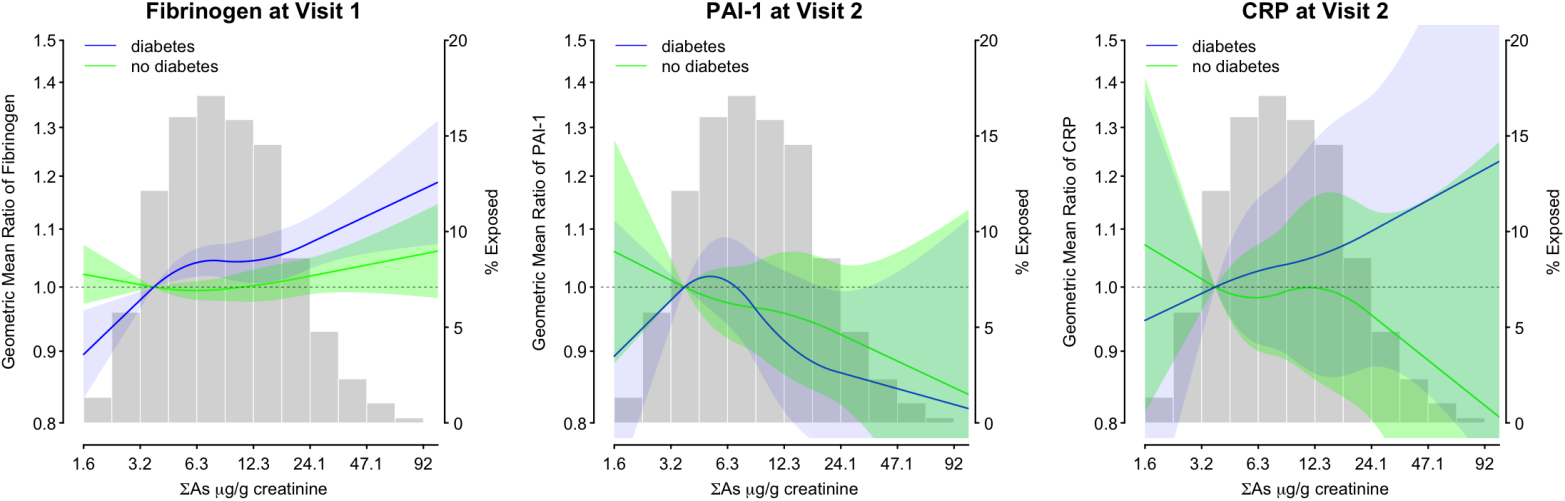
Geometric mean ratios of fibrinogen, PAI-1, and CRP in relation to urine arsenic in the SHS main cohort by diabetes status. Lines represent the geometric mean ratio (GMR) of baseline fibrinogen (left panel), PAI-1 at Visit 2 (center panel), or CRP at Visit 2 (right panel), by log-transformed urine arsenic concentrations (ΣAs, μg/g creatinine), with the 10th percentile (3.6 μg/g creatinine) as the reference. The GMR of baseline fibrinogen concentrations are from a linear mixed model and the GMR of Visit 2 PAI-1, and CRP concentrations are from a linear regression (see statistical methods for details). Arsenic was modeled using restricted quadratic splines of log-transformed urine arsenic (knots at the 10^th^, 50^th^, 90^th^ percentiles; 3.6, 8.4, and 22.4 μg/g creatinine, respectively). Models were fully-adjusted for all potential confounders in Model 2 (age, sex, education (no, some, or finished high school), smoking (never, former, current), alcohol drinking (never, former, current), BMI (kg/m^2^), LDL cholesterol (mg/dL), hypertension (yes/no), eGFR (mL/min/1.73 m^2^), and study center (AZ, OK, ND/SD).

A comparison of the 75^th^ to the 25^th^ percentile (14.3 vs. 5.1 μg/g creatinine) of baseline urine arsenic was associated with higher baseline fibrinogen concentrations among participants with diabetes (GMR: 1.05, 95% CI: 1.02, 1.07) but not among those without diabetes (GMR: 1.01, 95% CI: 0.99, 1.02) after adjusting for age, sex, education, smoking, alcohol drinking, BMI, LDL cholesterol, hypertension, eGFR, and study center (**Table 3**, Model 2; **Fig 1**). This association was statistically significantly different by diabetes status (p-interaction = 0.014 for log-transformed arsenic concentrations). Overall, and among those with diabetes, the association was attenuated and no longer significant after further adjustment for both albuminuria and hemoglobin A1c (**Table 3**, Model 3). In models where baseline urine arsenic treated as restricted quadratic splines of log-transformed urine arsenic, we found no statistical evidence of a non-linear association with baseline fibrinogen (**Table 3**).

**Table 3:** Geometric mean ratios (95% confidence interval) of baseline fibrinogen, Visit 2 PAI-1, and Visit 2 CRP by baseline urine arsenic concentrations by diabetes status in the Strong Heart Study main cohort.

		Urine Arsenic (ΣAs, μg/g creatinine)						
		Quartiles				Log-transformed 75^th^ vs. 25^th^ percentile ^a^		Quadratic Splines ^b^
		Q1	Q2	Q3	Q4			
	Median:	4.7	6.3	9.9	16.8	14.3 vs. 5.1	p-value	p-value
**Baseline Fibrinogen**								
Overall	Model 1 ^c^	1 (Ref)	1.02 (0.99, 1.04)	1.01 (0.99, 1.04)	**1.06 (1.04, 1.08)**	**1.03 (1.02, 1.04)**	**<0.001**	0.22
(N = 2700)	Model 2 ^d^	1 (Ref)	1.02 (0.99, 1.04)	1.01 (0.99, 1.04)	**1.05 (1.02, 1.07)**	**1.03 (1.01, 1.04)**	**<0.001**	0.43
	Model 3 ^e^	1 (Ref)	1.01 (0.99, 1.04)	1.01 (0.99, 1.03)	1.02 (0.99, 1.05)	1.01 (0.99, 1.03)	0.08	0.88
	Model 4 ^f^	1 (Ref)	1.02 (1.00, 1.04)	1.02 (0.99, 1.04)	1.06 (1.03, 1.08)	1.03 (1.02, 1.04)	<0.001	0.53
Without Diabetes	Model 1 ^c^	1 (Ref)	1.01 (0.99, 1.04)	0.99 (0.97, 1.02)	1.02 (0.99, 1.05)	1.01 (0.99, 1.02)	0.40	0.16
(N = 1145)	Model 2 ^d^	1 (Ref)	1.01 (0.99, 1.04)	1.00 (0.97, 1.03)	1.02 (0.99, 1.06)	1.01 (0.99, 1.02)	0.25	0.27
	Model 3 ^e^	1 (Ref)	1.01 (0.98, 1.04)	1.00 (0.97, 1.03)	1.01 (0.98, 1.05)	1.01 (0.99, 1.02)	0.50	0.23
With Diabetes	Model 1 ^c^	1 (Ref)	1.03 (0.99, 1.07)	**1.05 (1.01, 1.09)**	**1.10 (1.06, 1.14)**	**1.06 (1.04, 1.08)**	**<0.001**	0.24
(N = 1555)	Model 2 ^d^	1 (Ref)	1.03 (0.99, 1.07)	**1.04 (1.00, 1.09)**	**1.07 (1.03, 1.12)**	**1.05 (1.02, 1.07)**	**<0.001**	0.10
	Model 3 ^e^	1 (Ref)	1.02 (0.98, 1.06)	1.03 (0.99, 1.07)	1.04 (0.99, 1.08)	1.03 (0.99, 1.05)	0.06	0.24
**Visit 2 PAI-1**								
Overall	Model 1 ^c^	1 (Ref)	0.96 (0.89, 1.04)	**0.83 (0.77, 0.90)**	**0.80 (0.74, 0.86)**	**0.87 (0.84, 0.91)**	**<0.001**	0.35
(N = 1984)	Model 2 ^d^	1 (Ref)	0.99 (0.92, 1.07)	**0.92 (0.84, 1.00)**	0.91 (0.83, 1.00)	**0.94 (0.90, 0.99)**	**0.01**	0.61
	Model 3 ^e^	1 (Ref)	1.00 (0.92, 1.08)	0.90 (0.83 0.98)	0.92 (0.83, 1.01)	**0.94 (0.89, 0.99)**	**0.01**	0.68
	Model 4 ^f^	1 (Ref)	1.00 (0.92, 1.08)	0.92 (0.85, 1.00)	0.93 (0.84, 1.02)	0.95 (0.90 1.00)	0.033	0.61
Without Diabetes	Model 1 ^c^	1 (Ref)	0.93 (0.83, 1.04)	**0.81 (0.72, 0.92)**	**0.78 (0.68, 0.89)**	**0.87 (0.81, 0.93)**	**<0.001**	0.74
(N = 899)	Model 2 ^d^	1 (Ref)	0.96 (0.86, 1.08)	0.90 (0.79, 1.01)	0.89 (0.77, 1.03)	0.94 (0.87, 1.01)	0.10	0.95
	Model 3 ^e^	1 (Ref)	0.96 (0.85, 1.07)	0.89 (0.79, 1.01)	0.88 (0.76, 1.02)	0.94 (0.87, 1.01)	0.09	0.85
With Diabetes	Model 1 ^c^	1 (Ref)	0.98 (0.88, 1.09)	**0.84 (0.76, 0.94)**	**0.81 (0.73, 0.90)**	**0.88 (0.83, 0.93)**	**<0.001**	0.11
(N = 1085)	Model 2 ^d^	1 (Ref)	1.02 (0.92, 1.13)	0.93 (0.83, 1.05)	0.94 (0.84, 1.07)	0.95 (0.89, 1.01)	0.12	0.22
	Model 3 ^e^	1 (Ref)	1.02 (0.92, 1.13)	0.93 (0.83, 1.05)	0.94 (0.83, 1.07)	0.95 (0.89, 1.01)	0.14	0.16
**Visit 2 CRP**								
Overall	Model 1 ^c^	1 (Ref)	1.04 (0.92, 1.17)	1.10 (0.97, 1.24)	1.05 (0.93, 1.19)	1.05 (0.98, 1.11)	0.18	0.68
(N = 1984)	Model 2 ^d^	1 (Ref)	1.01 (0.90, 1.14)	1.03 (0.91, 1.17)	0.97 (0.84, 1.11)	1.00 (0.93, 1.08)	0.99	0.86
	Model 3 ^e^	1 (Ref)	0.99 (0.87, 1.12)	1.02 (0.90, 1.17)	0.95 (0.82, 1.10)	0.99 (0.92, 1.07)	0.82	0.77
	Model 4 ^f^	1 (Ref)	1.02 (0.91, 1.15)	1.04 (0.92, 1.19)	0.99 (0.86, 1.15)	1.02 (0.94 1.10)	0.68	0.84
Without Diabetes	Model 1 ^c^	1 (Ref)	1.02 (0.86, 1.21)	1.03 (0.87, 1.22)	0.96 (0.79, 1.16)	1.02 (0.92, 1.12)	0.73	0.88
(N = 899)	Model 2 ^d^	1 (Ref)	0.98 (0.83, 1.17)	0.94 (0.78, 1.12)	0.83 (0.67, 1.03)	0.95 (0.85, 1.06)	0.37	0.94
	Model 3 ^e^	1 (Ref)	0.97 (0.82, 1.16)	0.93 (0.78, 1.12)	0.82 (0.66, 1.01)	0.94 (0.85, 1.05)	0.31	0.99
With Diabetes	Model 1 ^c^	1 (Ref)	1.06 (0.90, 1.26)	1.14 (0.97, 1.35)	1.10 (0.94, 1.30)	1.05 (0.96, 1.14)	0.25	0.66
(N = 1085)	Model 2 ^d^	1 (Ref)	1.05 (0.89, 1.24)	1.11 (0.93, 1.33)	1.07 (0.88, 1.30)	1.03 (0.93, 1.14)	0.57	0.76
	Model 3 ^e^	1 (Ref)	1.05 (0.89, 1.24)	1.10 (0.92, 1.32)	1.04 (0.85, 1.27)	1.02 (0.92, 1.13)	0.59	0.58

Notes:

Q1, 1^st^ quartile; Q2, 2^nd^ quartile; Q3, 3^rd^ quartile; Q4, 4^th^ quartile; ΣAs, Sum of inorganic arsenic and methylated species in urine; PAI-1, Plasminogen activator inhibitor-1; CRP, C-reactive protein; Ref, Reference.

^a^ Geometric mean ratio comparing the 75^th^ percentile to the 25^th^ percentile of urine arsenic, estimated by multiplying the coefficient of log-transformed arsenic concentrations by the difference between the 75^th^ and 25^th^ percentiles on the log scale.

^b^ P-value from a Wald test that the two non-linear restricted quadratic spline coefficients are different from zero. Restricted quadratic splines were created from log-transformed arsenic concentrations, with knots at the 10^th^, 50^th^, and 90^th^ percentiles.

^c^ Model 1 adjusted for age, sex, and education (no, some, or finished high school), smoking (never, former, current), and alcohol drinking (never, former, current), body mass index (kg/m^2^), LDL cholesterol (mg/dL), hypertension (yes/no), and eGFR (mL/min/1.73 m^2^). Overall models (not stratified by diabetes status) were also adjusted for diabetes status.

^d^ Model 2 was further adjusted for study center (Arizona, Oklahoma, North and South Dakota).

^e^ Model 3 was further adjusted for albuminuria (ACR <30 mg/g, >30 to <300 mg/g, and ≥300 mg/g) and hemoglobin A1c (%). Hemoglobin A1c was measured in 93% of participants.

^f^ Model 4 was adjusted Model 2 variables without adjustment for diabetes.

In models examining the association between arsenic metabolism (urine %iAs, %MMA, and %DMA) and baseline plasma fibrinogen, we found no association with %iAs (GMR: 1.01, 95% CI: 1.00, 1.01; p = 0.26), a positive association with %MMA (GMR: 1.03, 95% CI: 1.01, 1.04), and an inverse association with %DMA (GMR: 0.98, 95% CI: 0.97, 0.99). There was no evidence of an interaction between any of the urine methylation markers and diabetes status (all p-interaction <0.05).

### Associations between baseline urine arsenic and plasma PAI-1 and CRP at Visit 2 in the SHS main cohort

In fully adjusted models, comparison of the 75^th^ to the 25^th^ percentile (14.3 vs. 5.1 μg/g creatinine) of baseline urine arsenic was not significantly associated with Visit 2 PAI-1 when stratifying in those with or without diabetes (GMR without diabetes: 0.94, 95% CI: 0.87, 1.01; GMR with diabetes: 0.95, 95% CI: 0.89, 1.01) and there was no significant difference in the association between arsenic and PAI-1 by diabetes status (p-interaction = 0.66) (**Table 3**, Model 2; **Fig 1**). Overall, the corresponding GMR of Visit 2 PAI-1 concentrations (GMR: 0.94, 95% CI: 0.89, 0.99) by arsenic levels was statistically significant (**Table 3,** Model 2). For CRP, a corresponding difference in baseline urine arsenic was not associated with Visit 2 CRP concentrations either overall (GMR: 1.00, 95% CI: 0.93, 1.08) or stratified by diabetes status (GMR without diabetes: 0.95, 95% CI: 0.85, 1.06; GMR with diabetes: 1.03, 95% CI: 0.93, 1.14; p-interaction = 0.66) (**Table 3**, Model 3; **Fig 1**). Additional adjustment for time between baseline and Visit 2 did not affect the associations. Results were consistent using arsenic quartiles created from the set of participants with complete data at baseline, and when stratifying by baseline diabetes. In post-hoc subgroup analyses, we found some evidence for effect modification of the association between urine arsenic and CRP concentrations by sex (**S2 Table**).

For plasma PAI-1, we found no association with %iAs (GMR: 1.01, 95% CI: 1.00, 1.01; p = 0.080), an inverse association with %MMA (GMR: 0.94, 95% CI: 0.91, 0.98), and a positive association with %DMA (GMR: 1.02, 95% CI: 1.06, 1.10). There was no evidence of an interaction between any of the urine methylation markers and diabetes status (all p-interaction >0.05).

### Cross-sectional association between baseline urine arsenic and fibrinogen, PAI-1, and CRP in SHFS participants without diabetes

Among 1901 participants without diabetes at the SHFS baseline (Visit 3 pilot/Visit 4), the median (IQR) age was 36 (24, 47) years, 60% were female, and 70% had finished high school (**S3 Table**). The overall median (IQR) of urine arsenic was 4.3 (2.9, 7.1) μg/g creatinine. The median (IQR) baseline concentrations of fibrinogen, PAI-1, and CRP were 359 (311, 416) mg/dL, 45 (27, 69) ng/mL, and 3.2 (1.2, 6.9) mg/L, respectively. Participants with higher urine arsenic were older, more likely to be female, had less education, were more likely to smoke, and had a higher prevalence of albuminuria. We present the median concentrations of fibrinogen, PAI-1, and CRP in relation to selected participant characteristics in **S4 Table**.

After adjusting for age, sex, education, smoking, alcohol drinking, BMI, LDL cholesterol, hypertension, eGFR, and study center, there was no association between a difference in the 75^th^ versus the 25^th^ percentile of urine arsenic and fibrinogen (GMR: 0.99, 95% CI: 0.98, 1.01), PAI-1 (GMR: 1.01, 95% CI: 0.97, 1.06), or CRP (GMR: 0.95, 95% CI: 0.87, 1.02) among SHFS participants without diabetes (**S5 Table**, Model 2; **S3 Fig**). In post-hoc subgroup analyses, we found some evidence for effect modification of the association between arsenic and fibrinogen by LDL cholesterol and PAI-1 and CRP concentrations by study site (**S6 Table**).

## Discussion

In the SHS main cohort, a population of adult men and women exposed to low-moderate levels of arsenic in drinking water (<100 μg/L), we identified a positive association with plasma fibrinogen limited to participants with diabetes and an inverse association with plasma PAI-1 in relation to baseline urine arsenic concentrations. Further adjustment for albuminuria and hemoglobin A1c, which may act as confounders or mediators of the association between arsenic and CVD [58, 59], attenuated the association with fibrinogen but did not change the association with PAI-1. We found no associations between baseline urine arsenic and plasma CRP in the SHS main cohort, and no associations between baseline urine arsenic and fibrinogen, PAI-1, and CRP in a secondary analysis of SHFS participants without diabetes.

Arsenic may dysregulate one or multiple pathways related to CVD development and progression. As reviewed recently by Wu *et al*., the strongest epidemiologic evidence for an association between chronic arsenic exposure and subclinical CVD endpoints comes from studies of subclinical atherosclerosis, QT interval prolongation, and circulating markers of endothelial dysfunction, particularly soluble intercellular and vascular cell adhesion molecules (sICAM-1 and sVCAM-1) and most studies have been conducted among populations exposed to arsenic in drinking water above 100 μg/L [62]. To our knowledge, this is the first general population epidemiologic study to examine the association between chronic arsenic exposure and plasma fibrinogen and the first epidemiologic study to examine low-moderate arsenic exposure (<100 μg/L in drinking water) and PAI-1 or CRP concentrations. A small clinical study (36 cases and 100 controls) found higher levels of platelet aggregation and coagulation factors, including plasma fibrinogen, in subjects with Blackfoot disease, a peripheral vascular disease related to endemic high arsenic exposure in Taiwan [32].

Higher plasma fibrinogen concentrations indicate impaired fibrinolysis (i.e., an increased risk of thrombosis), but fibrinogen is also an acute phase protein that is upregulated downstream of the cytokine-driven inflammatory cascade [63]. A meta-analysis of individual participant data from prospective cohort studies found that a difference of 1 g/L of fibrinogen was associated with an almost two-fold increase in the risk of CHD, stroke, and vascular mortality in CVD-free individuals, with no evidence of differential risk by diabetes status [19]. In the SHS, fibrinogen was associated with a higher risk of incident non-fatal and fatal CVD events [64, 65] but not with ischemic stroke [66]. It is uncertain whether fibrinogen is causally associated with CVD, and Mendelian randomization studies have not supported a causal role for fibrinogen in CVD [67]. Plasma fibrinogen levels increase strongly with age, and are associated with traditional CVD risk factors like lipids, obesity, and diabetes [68, 69]. We adjusted for age in multivariable regression models.

Our finding of a differential association between arsenic exposure and fibrinogen concentrations by diabetes status is consistent with our previous findings in the SHS main cohort that the association between baseline urine arsenic and incident CVD and CHD was stronger among participants with diabetes [12]. The etiologic pathways proposed for the cardiovascular effects of chronic arsenic exposure may share commonalities with the pathophysiology of diabetic vasculopathy. For example, diabetes has been linked to subclinical atherosclerosis, such as increases in carotid intima-media thickness [70], endothelial dysfunction [71], and chronic inflammation [72].

The explanation for our observed inverse association between arsenic and PAI-1 concentrations in the SHS main cohort, particularly in contrast to the null association in SHFS participants without diabetes, is unclear. We were surprised to find that PAI-1 was inversely associated with several CVD risk factors in univariate analyses, including age and eGFR in both participants with and without diabetes, and with SBP, hemoglobin A1c, and education in participants with diabetes. Levels of PAI-1 may reflect a mixture of inflammation, metabolic control, and neurohormonal activation, all of which may contribute to CVD risk [73, 74]. PAI-1 concentrations were associated with incident CHD in the Framingham Study [21], but were not associated with incident CVD in the SHS main cohort [64]. Previous epidemiologic studies of chronic arsenic exposure, albeit at high levels of arsenic in drinking water (>100 μg/L), have all found positive associations with plasma PAI-1 concentrations [28, 29]. In Taiwan, 28 Blackfoot disease patients had higher PAI-1 levels compared to age-matched controls [28]. In a sample of 668 HEALS study participants in Bangladesh, higher water arsenic was associated with higher PAI-1 concentrations, with a stronger association among participants with a BMI above 19.1 mg/kg^2^ [29]. Further, cultured human microvascular endothelial cells exposed to 50 to 500 μg/L sodium arsenite had higher PAI-1 levels and higher PAI-1 activity compared to controls [33].

CRP is commonly considered a sensitive biomarker of nonspecific systemic inflammation, but it may also have pleotropic effects in atherosclerosis through its effects on adhesion molecule expression, fibrinolysis, and endothelial dysfunction [75]. Current evidence suggests that CRP is unlikely to play a causal role in CVD [76]. In the SHS main cohort, baseline CRP was associated with incident CVD events, although the association was limited to participants without diabetes [36]. Small cross-sectional studies of populations exposed to high levels of arsenic exposure in drinking water (>100 μg/L) in Bangladesh have found a positive association with CRP [30, 31]. In human hepatic cells, relatively low levels of arsenic (0.13 to 0.67 μM of sodium arsenite, equivalent to 17 to 87 μg/L) resulted in significantly higher CRP expression and secretion compared to controls and mice exposed to 100 μg/L of sodium arsenite in drinking water for six months had higher CRP expression in liver cells compared to controls [34].

We observed contrasting associations between arsenic methylation and plasma biomarkers of thrombosis and inflammation. Higher %MMA was positively associated and %DMA was negatively associated with baseline fibrinogen, whereas higher %MMA was negatively associated and %DMA was positively associated with Visit 2 PAI-1. These results suggest that lower arsenic metabolism is associated with higher fibrinogen but that higher or more complete arsenic methylation is associated with PAI-1. Although methylation was initially thought to reduce arsenic toxicity, the association between arsenic metabolism and health effects has been found to be complex and may differ across arsenic-related disease outcomes. In a recent systematic review, most studies observed that higher %MMA and lower %DMA was associated with CVD outcomes, whereas lower %MMA and higher %DMA was often associated with diabetes and metabolic syndrome [77]. PA1-1 is generally more strongly related to obesity, insulin resistance, and diabetes compared to fibrinogen [74], although the relationship between the inflammatory and fibrinolytic systems, observed as plasma fibrinogen and PAI-1, and CVD and diabetes is complex. Thus, our results of a differential association between urine arsenic methylation markers and fibrinogen compared to PAI-1 are consistent with this general pattern of lower methylation associated with CVD outcomes and higher methylation related to diabetes-related outcomes.

The high-quality measurement of speciated arsenic in urine is a major strength of this study and is particularly useful in a population with low-moderate arsenic exposure in drinking water where diet can contribute substantially to overall exposure [40]. The SHS had high quality data collection methods, information on important metabolic and lifestyle CVD factors, and little loss to follow-up or missing data. In the SHS main cohort, plasma fibrinogen was measured at multiple visits, allowing for the examination of both cross-sectional and longitudinal associations. Although plasma fibrinogen, PAI-1, and CRP vary diurnally [78–80], the influence of circadian variation is likely minimized in the SHS because all biological samples were collected in the morning.

This analysis also had some limitations. Urine arsenic is likely a good biomarker of chronic exposure in this population because concentrations of urine arsenic remained stable over 10 years in the SHS [12], drinking water arsenic levels tend to be relatively constant over time [81], and the SHS participants have low residential mobility; however, the half-lives of urine arsenic species are relatively short (e.g., days to weeks). Measurements of plasma fibrinogen, PAI-1, and CRP taken several years apart may vary due to measurement error, chronic disease, aging, or changes in other CVD risk factors. PAI-1 and CRP were only available at Visit 2, and the association between baseline arsenic and Visit 2 CRP and PAI-1 concentrations may have been different if these biomarkers were measured at baseline. Due to the observational nature of the study, there is the possibility of selection bias and residual confounding. Residual confounding by socioeconomic status, geographic factors, or other factors is possible, although our results were robust to adjustment for traditional CVD risk factors, education, and study site. While collider stratification bias is a potential concern in epidemiological studies, we did not see major differences in models with and without adjustment for diabetes and thus we believe it is unlikely that diabetes is acting a collider. We cannot rule out reverse causation, especially considering prothrombotic and inflammatory factors are elevated in kidney disease [82], and urine arsenic concentrations may be affected by kidney function [83]. Absolute levels of fibrinogen and PAI-1 were generally higher than in other studies [22, 36, 64, 84], likely reflecting the higher prevalence of obesity, diabetes, and insulin resistance in the SHS. Our results may not be generalizable to other populations with different CVD risk factor profiles. Our secondary analysis using SHFS data was limited to individuals without diabetes because urine arsenic has now been measured only in participants without diabetes at baseline.

About 1.8 million United States residents are exposed to arsenic in public drinking water above the EPA standard of 10 μg/L [1] and approximately three million are exposed to arsenic in private wells above 10 μg/L [2–4]. Examining the association between arsenic and subclinical CVD endpoints can help support arsenic risk assessment and drinking water policy by providing evidence for pathophysiological pathways that link arsenic and clinical CVD endpoints. Emerging untargeted strategies, such as metabolomics and epigenome-wide association studies, could help identify mechanistic pathways for arsenic-related CVD.

In summary, we identified a positive cross-sectional association between low-moderate chronic arsenic exposure and plasma fibrinogen concentrations in participants with diabetes and an unexpected inverse association with plasma PAI-1 in the Strong Heart Study. Additional research is needed to understand how environmental exposures, such as arsenic, can increase the risk of cardiovascular disease in the presence of diabetes. Future studies, particularly at low-moderate levels of arsenic exposure, are needed to confirm these associations and should investigate additional subclinical markers that could explain the association between chronic arsenic exposure and CVD.

## Supporting information

S1 Fig Inclusion criteria for analyses in the Strong Heart Study main cohort (Visit 1, 2, and 3). (PDF)

S2 Fig Inclusion criteria for analyses in the Strong Heart Study family study participants without diabetes (Visit 3 pilot/Visit 4 baseline). (PDF)

S3 Fig Geometric mean ratios of baseline fibrinogen, PAI-1, and CRP in Strong Heart Family Study (SHFS) participants without diabetes (Visit 3 pilot/Visit 4) in relation to baseline urine arsenic concentrations. (DOCX)

S1 Table Selected characteristics of SHS main cohort participants at baseline (Visit 1) by diabetes status and quartiles of urine arsenic. (DOCX)

S2 Table Geometric mean ratios (95% confidence intervals) for baseline fibrinogen, Visit 2 PAI-1, and Visit 2 CRP in relation to baseline urine arsenic in SHS main cohort participants by baseline participant characteristics. (DOCX)

S3 Table Selected characteristics of Strong Heart Family Study (SHFS) participants without diabetes at baseline (Visit 3 pilot/Visit 4) by quartiles of urine arsenic. (DOCX)

S4 Table Median concentrations of plasma fibrinogen, PAI-1, and CRP in Strong Heart Family Study (SHFS) participants without diabetes by participant characteristics. (DOCX)

S5 Table Adjusted geometric mean ratios (95% confidence intervals) of baseline (Visit 3 pilot/Visit 4) plasma fibrinogen, PAI-1, and CRP concentrations in Strong Heart Family Study (SHFS) participants without diabetes by urine arsenic concentrations. (DOCX)

S6 Table Geometric mean ratios (95% confidence intervals) for baseline fibrinogen, Visit PAI-1, and Visit 2 CRP in relation to baseline urine arsenic in SHFS participants without diabetes by baseline participant characteristics. (DOCX)
