# Supplementary material for: Low-moderate urine arsenic and biomarkers of thrombosis and inflammation in the Strong Heart Study
Source: PLoS One. 2017 Aug 3;12(8):e0182435. doi: 10.1371/journal.pone.0182435 (PMC5542675; doi:10.1371/journal.pone.0182435)

S1 Fig. Inclusion Criteria for Analyses in the Strong Heart Study Main Cohort (Visit 1, 2, and 3)

SHS, Strong Heart Study; CVD, Cardiovascular disease; BMI, Body mass index; LDL, Low-density lipoprotein; eGFR, estimated glomerular filtration rate
* Analyses include 12 SHS communities with available data and permission to conduct the study.

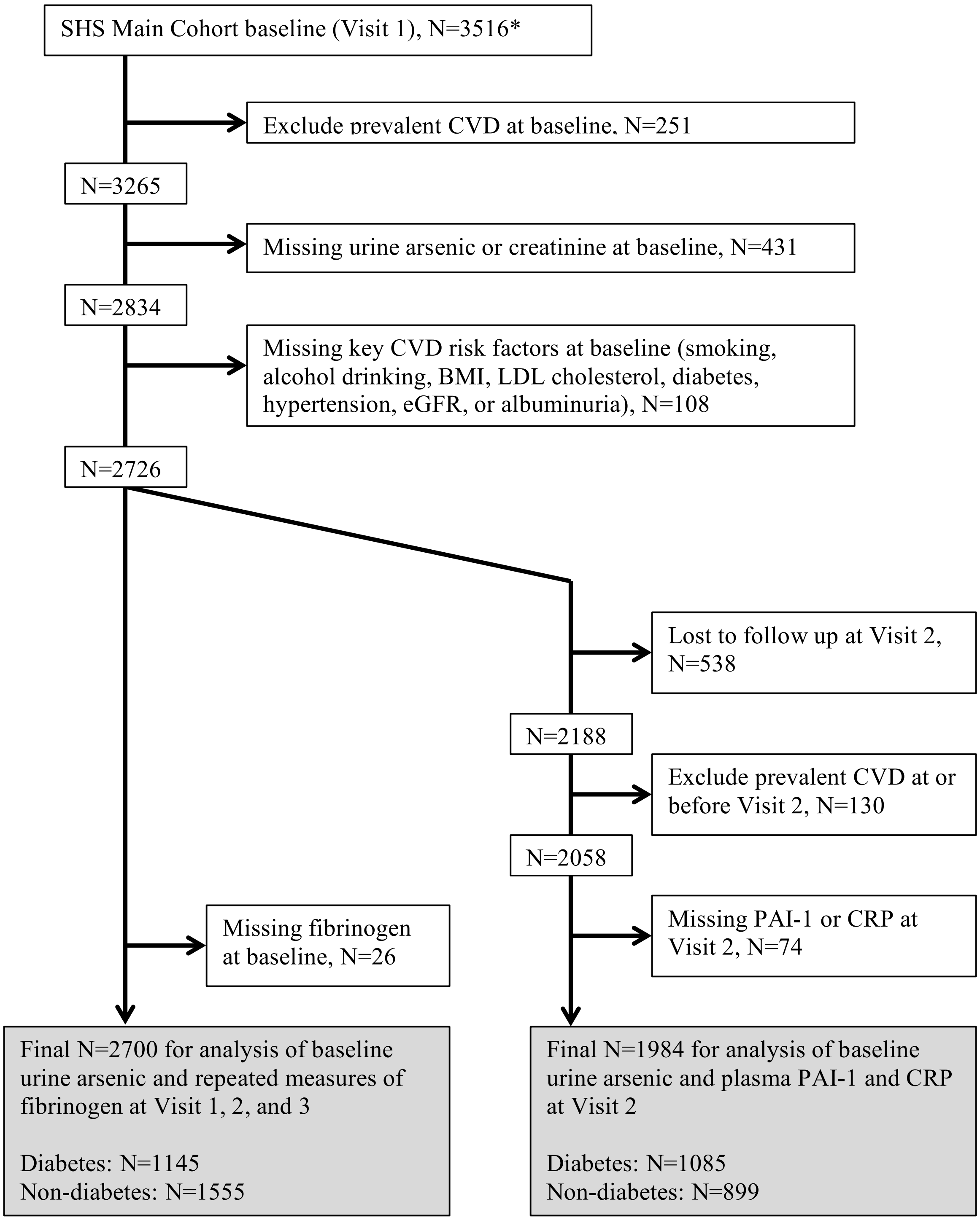

Supplement: S1 Fig — (PDF) [file pone.0182435.s001.pdf]
