# Supplementary material for: Low-moderate urine arsenic and biomarkers of thrombosis and inflammation in the Strong Heart Study
Source: PLoS One. 2017 Aug 3;12(8):e0182435. doi: 10.1371/journal.pone.0182435 (PMC5542675; doi:10.1371/journal.pone.0182435)

S2 Fig. Inclusion Criteria for Analyses in the Strong Heart Study Family Study Participants Without Diabetes (Visit 3 pilot/Visit 4 Baseline)

SHS, Strong Heart Study; CVD, Cardiovascular disease; BMI, Body mass index; LDL, Low-density lipoprotein; eGFR, estimated glomerular filtration rate

* Analyses include 12 SHS communities with available data and permission to conduct the study.

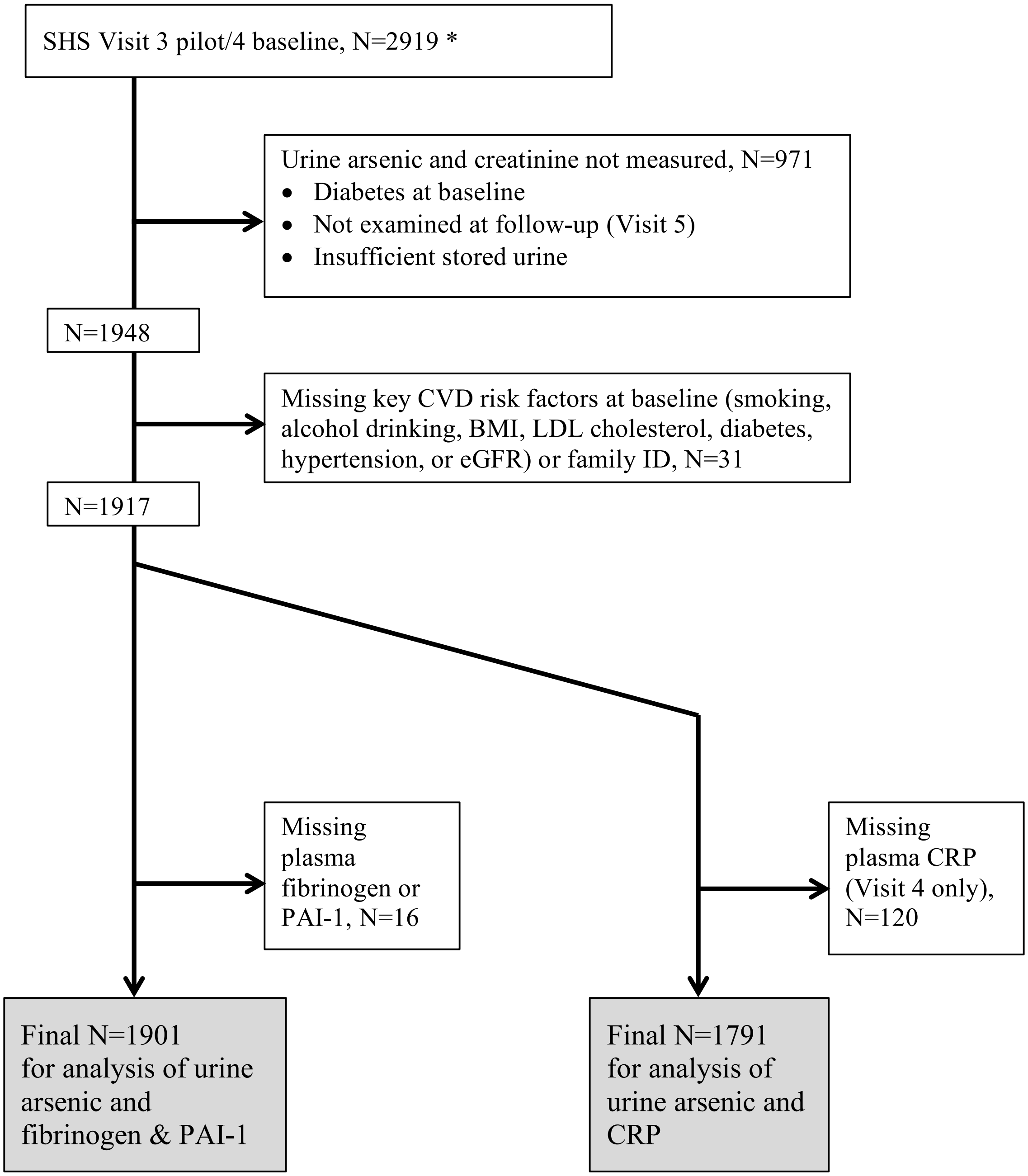

Supplement: S2 Fig — (PDF) [file pone.0182435.s002.pdf]
