# Supplementary material for: Low-moderate urine arsenic and biomarkers of thrombosis and inflammation in the Strong Heart Study
Source: PLoS One. 2017 Aug 3;12(8):e0182435. doi: 10.1371/journal.pone.0182435 (PMC5542675; doi:10.1371/journal.pone.0182435)
Supplement: S3 Fig — (DOCX) [file pone.0182435.s003.docx]

# S3 Fig. Geometric Mean Ratios of Baseline Fibrinogen, PAI-1, and CRP in Strong Heart Family Study (SHFS) Participants without Diabetes (Visit 3 pilot/Visit 4) in relation to Baseline Urine Arsenic Concentrations

Lines represent the geometric mean ratio (GMR) of plasma fibrinogen, PAI-1, or CRP, by log-transformed urine arsenic concentrations, with the 10th percentile of arsenic (2.2 µg/g creatinine) as the reference. Shaded regions represent the upper and lower boundaries of the 95^th^ confidence limits of the GMR. GMR estimated from linear mixed models with a random intercept for family to account for potential correlation within families. Arsenic was modeled using restricted quadratic splines of log-transformed urine arsenic (knots at the 10^th^, 50^th^, 90^th^ percentiles; 2.2, 4.3, and 11.6 µg/g creatinine, respectively). Models were fully adjusted for all potential confounders in Model 2 (age, sex, education (no, some, or finished high school), smoking (never, former, current), alcohol drinking (never, former, current), BMI (kg/m^2^), LDL cholesterol (mg/dL), hypertension (yes/no), and eGFR (mL/min/1.73 m^2^), and study center (AZ, OK, ND/SD).
