# Supplementary material for: Low-moderate urine arsenic and biomarkers of thrombosis and inflammation in the Strong Heart Study
Source: PLoS One. 2017 Aug 3;12(8):e0182435. doi: 10.1371/journal.pone.0182435 (PMC5542675; doi:10.1371/journal.pone.0182435)
Supplement: S1 Table — (DOCX) [file pone.0182435.s004.docx]

# S1 Table. Selected Characteristics of SHS Main Cohort Participants at Baseline (Visit 1) by Diabetes Status and Quartiles of Urine Arsenic

|  | **Participants Without Diabetes (N=1555)** | | | | | **Participants With Diabetes (N=1145)** | | | | |
| --- | --- | --- | --- | --- | --- | --- | --- | --- | --- | --- |
|  | **Overall** | Σ**As Q1**  **N=436** | Σ**As Q2**  **N=409** | Σ**As Q3**  **N=398** | Σ**As Q4**  **N=312** | **Overall** | Σ**As Q1**  **N=240** | Σ**As Q2**  **N=266** | Σ**As Q3**  **N=279** | Σ**As Q4**  **N=360** |
| Mean | 12.7 | 3.7 | 6.7 | 11.1 | 24.3 | 10.7 | 3.8 | 6.6 | 11.0 | 25.5 |
| Median | 9.6 | 3.8 | 6.7 | 11.0 | 20.7 | 7.7 | 3.8 | 6.5 | 10.9 | 20.1 |
| Range | 1.9, 179.9 | 1.9, 5.1 | 5.2, 8.4 | 8.4, 14.3 | 14.4, 179.9 | 1.7, 123.6 | 1.7, 5.1 | 5.2, 8.4 | 8.4, 14.3 | 14.3, 123.6 |
| Age, years | 56 (50, 63) | 57 (50, 64) | 56 (50, 65) | 55 (50, 62) | 56 (50, 63) | 54 (48, 61) | 53 (48, 61) | 54 (48, 61) | 54 (49, 61) | 54 (49, 62) |
| Female, % | 871 (56%) | 202 (46%) | 246 (60%) | 233 (59%) | 190 (61%) | 729 (64%) | 130 (54%) | 167 (63%) | 181 (65%) | 251 (70%) |
| Finished high school, % | 975 (63%) | 311 (71%) | 276 (67%) | 230 (58%) | 158 (51%) | 627 (55%) | 158 (66%) | 162 (61%) | 149 (53%) | 158 (44%) |
| Current smoker | 653 (42%) | 159 (36%) | 158 (39%) | 174 (44%) | 162 (52%) | 367 (32%) | 74 (31%) | 90 (34%) | 100 (36%) | 103 (29%) |
| Current drinker | 751 (48%) | 165 (38%) | 184 (45%) | 211 (53%) | 191 (61%) | 406 (35%) | 72 (30%) | 78 (29%) | 122 (44%) | 134 (37%) |
| BMI, kg/m^2^ | 29 (25, 32) | 29 (26, 33) | 29 (26, 32) | 29 (25, 32) | 27 (24, 31) | 31 (28, 36) | 32 (28, 36) | 32 (28, 36) | 31 (28, 35) | 30 (27, 35) |
| Hypertension, % | 433 (28%) | 122 (28%) | 125 (31%) | 114 (29%) | 72 (23%) | 527 (46%) | 129 (54%) | 123 (46%) | 116 (42%) | 159 (44%) |
| Hemoglobin A1c, % | 5.1 (4.7, 5.4) | 5.1 (4.8, 5.4) | 5.1 (4.8, 5.4) | 5.0 (4.7, 5.4) | 5.0 (4.7, 5.4) | 7.6 (5.9, 9.9) | 6.7 (5.6, 8.8) | 7.0 (5.9, 9.6) | 7.6 (5.9, 10.0) | 8.6 (6.4, 11.1) |
| LDL cholesterol, mg/dL | 121 (100, 143) | 123 (102, 146) | 122 (101, 144) | 122 (101, 142) | 115 (94, 139) | 116 (95, 137) | 117 (97, 140) | 120 (98, 139) | 116 (95, 132) | 111 (90, 136) |
| eGFR, mL/min/1.73 m^2^ | 100 (92, 107) | 99 (90, 106) | 100 (93, 108) | 100 (91, 107) | 103 (95, 110) | 100 (90, 108) | 97 (85, 105) | 98 (85, 107) | 101 (92, 108) | 102 (93, 111) |
| Albuminuria, % | 143 (9%) | 24 (6%) | 37 (9%) | 37 (9%) | 45 (14%) | 486 (42%) | 78 (32%) | 94 (35%) | 106 (38%) | 208 (58%) |
| Post-menopause, % | 623 (72%) | 142 (70%) | 167 (68%) | 170 (73%) | 144 (76%) | 589 (81%) | 109 (84%) | 143 (86%) | 141 (78%) | 196 (78%) |
| Fibrinogen, mg/dL (V1) | 276 (238, 320) | 276 (234, 320) | 276 (238, 320) | 276 (236, 310) | 276 (244, 320) | 304 (254, 358) | 292 (257, 338) | 296 (252, 338) | 304 (249, 362) | 316 (264, 374) |
| Fibrinogen, mg/dL (V2) | 334 (295, 377) | 324 (289, 367) | 340 (298, 390) | 331 (293, 367) | 345 (307, 383) | 364 (320, 416) | 348 (300, 399) | 352 (320, 403) | 364 (317, 423) | 381 (335, 436) |
| Fibrinogen, mg/dL (V3) | 351 (304, 411) | 344 (294, 404) | 346 (301, 418) | 347 (307, 411) | 366 (315, 423) | 387 (327, 451) | 380 (315, 447) | 387 (330, 443) | 380 (327, 436) | 404 (341, 472) |
| PAI-1, ng/mL (V2) | 42 (29, 65) | 48 (32, 70) | 45 (30, 64) | 38 (26, 60) | 38 (25, 66) | 46 (31, 70) | 52 (38, 78) | 50 (34, 78) | 42 (30, 64) | 40 (29, 62) |
| CRP, mg/L (V2) | 3.5 (1.7, 6.0) | 3.3 (1.7, 5.5) | 3.5 (1.8, 6.0) | 3.7 (1.7, 6.1) | 3.2 (1.7, 6.5) | 4.5 (2.5, 8.4) | 3.8 (2.2, 6.9) | 4.6 (2.3, 8.2) | 4.9 (2.7, 9.4) | 4.5 (2.5, 8.4) |

Notes:

Albuminuria defined as an albumin: creatinine ratio >30 mg/g. Percentage of post-menopausal calculated for women only.
