# Supplementary material for: Low-moderate urine arsenic and biomarkers of thrombosis and inflammation in the Strong Heart Study
Source: PLoS One. 2017 Aug 3;12(8):e0182435. doi: 10.1371/journal.pone.0182435 (PMC5542675; doi:10.1371/journal.pone.0182435)
Supplement: S2 Table — (DOCX) [file pone.0182435.s005.docx]

# S2 Table. Geometric Mean Ratios (95% Confidence Intervals) for Baseline Fibrinogen, Visit 2 PAI-1, and Visit 2 CRP in Relation to Baseline Urine Arsenic in SHS Main Cohort Participants by Baseline Participant Characteristics

| **Participant**  **Characteristic** | **Fibrinogen** | | | **PAI-1** | | | **CRP** | | |
| --- | --- | --- | --- | --- | --- | --- | --- | --- | --- |
|  | **N** | **GMR (95% CI)** | **p-interaction** | **N** | **GMR (95% CI)** | **p-interaction** | **N** | **GMR (95% CI)** | **p-interaction** |
| Age |  |  |  |  |  |  |  |  |  |
| <55 y | 1382 | 1.03 (1.01, 1.05) |  | 1067 | 0.93 (0.88, 0.99) |  | 1067 | 1.00 (0.92, 1.10) |  |
| ≥55 y | 1318 | 1.02 (1.00, 1.04) | 0.59 | 917 | 0.94 (0.88, 1.00) | 0.86 | 917 | 0.98 (0.89, 1.08) | 0.68 |
| Sex |  |  |  |  |  |  |  |  |  |
| Men | 1100 | 1.03 (1.01, 1.05) |  | 749 | 0.93 (0.86, 0.99) |  | 749 | 1.09 (0.97, 1.21) |  |
| Women | 1600 | 1.02 (1.01, 1.04) | 0.53 | 1235 | 0.95 (0.9, 1.00) | 0.56 | 1235 | 0.96 (0.88, 1.04) | **0.045** |
| Education |  |  |  |  |  |  |  |  |  |
| <High school | 1098 | 1.02 (1.00, 1.04) |  | 763 | 0.95 (0.89, 1.02) |  | 763 | 1.00 (0.9, 1.11) |  |
| ≥High school | 1602 | 1.03 (1.01, 1.05) | 0.45 | 1221 | 0.93 (0.87, 0.98) | 0.51 | 1221 | 1.00 (0.91, 1.09) | 0.93 |
| BMI |  |  |  |  |  |  |  |  |  |
| <30 kg/m^2^ | 1420 | 1.02 (1.01, 1.04) |  | 1023 | 0.95 (0.89, 1.00) |  | 1023 | 1.03 (0.94, 1.13) |  |
| ≥30 kg/m^2^ | 1280 | 1.03 (1.01, 1.05) | 0.72 | 961 | 0.91 (0.85, 0.98) | 0.39 | 961 | 0.92 (0.83, 1.02) | 0.08 |
| LDL Cholesterol |  |  |  |  |  |  |  |  |  |
| <100 mg/dL | 739 | 1.01 (0.99, 1.03) |  | 526 | 0.99 (0.91, 1.07) |  | 526 | 0.95 (0.84, 1.07) |  |
| ≥100 mg/dL | 1961 | 1.03 (1.02, 1.05) | 0.09 | 1458 | 0.92 (0.88, 0.98) | 0.13 | 1458 | 1.02 (0.94, 1.11) | 0.28 |
| Hypertension |  |  |  |  |  |  |  |  |  |
| No | 1740 | 1.02 (1.00, 1.03) |  | 1308 | 0.94 (0.89, 1.00) |  | 1308 | 0.97 (0.89, 1.06) |  |
| Yes | 960 | 1.04 (1.02, 1.06) | 0.08 | 676 | 0.94 (0.87, 1.01) | 0.89 | 676 | 1.05 (0.94, 1.17) | 0.22 |
| eGFR |  |  |  |  |  |  |  |  |  |
| >60 ml/min/1.73m^2^ | 2611 | 1.02 (1.01, 1.04) |  | 1937 | 0.96 (0.91, 1.00) |  | 1937 | 0.99 (0.92, 1.07) |  |
| ≤60 ml/min/1.73m^2^ | 89 | 1.02 (0.97, 1.08) | 0.94 | 47 | 0.82 (0.67, 1.01) | 0.15 | 47 | 1.09 (0.79, 1.50) | 0.56 |
| Diabetes |  |  |  |  |  |  |  |  |  |
| No | 1555 | 1.01 (1.00, 1.03) |  | 899 | 0.93 (0.87, 0.99) |  | 899 | 0.99 (0.89, 1.09) |  |
| Yes | 1145 | 1.04 (1.02, 1.06) | **0.01** | 1085 | 0.95 (0.89, 1.01) | 0.66 | 1085 | 1.01 (0.92, 1.11) | 0.66 |
| Smoking |  |  |  |  |  |  |  |  |  |
| Never | 789 | 1.01 (1.00, 1.03) |  | 610 | 0.93 (0.87, 0.99) |  | 610 | 0.99 (0.89, 1.09) |  |
| Former | 891 | 1.03 (1.01, 1.05) |  | 666 | 0.95 (0.88, 1.03) |  | 666 | 0.97 (0.86, 1.09) |  |
| Current | 1020 | 1.03 (1.01, 1.05) | 0.84 | 708 | 0.95 (0.88, 1.03) | 0.15 | 708 | 0.97 (0.86, 1.09) | 0.80 |
| Alcohol Use |  |  |  |  |  |  |  |  |  |
| Never | 403 | 1.01 (1.00, 1.03) |  | 305 | 0.93 (0.87, 0.99) |  | 305 | 0.99 (0.89, 1.09) |  |
| Former | 1140 | 1.01 (0.98, 1.04) |  | 843 | 0.88 (0.79, 0.98) |  | 843 | 0.85 (0.72, 1.01) |  |
| Current | 1157 | 1.01 (0.98, 1.04) | 0.42 | 836 | 0.88 (0.79, 0.98) | 0.32 | 836 | 0.85 (0.72, 1.01) | 0.09 |

GMR, Geometric mean ratio; LDL, Low density lipoprotein; BMI, Body mass index; eGFR, estimated glomerular filtration rate
