# Supplementary material for: Low-moderate urine arsenic and biomarkers of thrombosis and inflammation in the Strong Heart Study
Source: PLoS One. 2017 Aug 3;12(8):e0182435. doi: 10.1371/journal.pone.0182435 (PMC5542675; doi:10.1371/journal.pone.0182435)
Supplement: S3 Table — (DOCX) [file pone.0182435.s006.docx]

# S3 Table. Selected Characteristics of Strong Heart Family Study (SHFS) Participants without Diabetes at Baseline (Visit 3 pilot/Visit 4) by Quartiles of Urine Arsenic

|  |  | | **Quartiles of Urine Arsenic (Inorganic & Methylated Metabolites)** | | | | |
| --- | --- | --- | --- | --- | --- | --- | --- |
|  | **Overall**  **N=1,901** | **Q1**  **N=478** | | **Q2**  **N=474** | **Q3**  **N=475** | **Q4**  **N=474** |  |
| **Mean (SD)** | **6.1 (8.2)** | **2.2 (0.5)** | | **3.6 (0.4)** | **5.4 (0.8)** | **13.1 (14.1)** | **p-** |
| **Median (IQR)** | **4.3 (2.9, 7.1)** | **2.3 (1.9, 2.6)** | | **3.6 (3.2, 3.9)** | **5.2 (4.8, 6.0)** | **10.3 (8.2, 13.6)** | **value** |
| **Range** | **0.4, 264.5** | **0.4, 2.9** | | **2.9, 4.3** | **4.3, 7.1** | **7.1, 264.5** |  |
| Age, years | 36 (24, 47) | 33 (22, 45) | | 35 (24, 47) | 38 (25, 48) | 38 (25, 49) | **0.004** |
| Female, % | 1143 (60%) | 275 (58%) | | 251 (53%) | 305 (64%) | 312 (66%) | **<0.001** |
| Finished high school, % | 1329 (70%) | 359 (75%) | | 349 (74%) | 333 (70%) | 288 (61%) | **<0.001** |
| Current smoker, % | 724 (38%) | 146 (31%) | | 173 (36%) | 195 (41%) | 210 (44%) | <**0.001** |
| Current drinker, % | 1201 (63%) | 306 (64%) | | 288 (61%) | 307 (65%) | 300 (63%) | 0.62 |
| BMI, mg/kg^2^ | 30 (26, 35) | 30 (25, 35) | | 29 (25, 34) | 30 (26, 35) | 30 (26, 36) | 0.12 |
| Hypertension, % | 424 (22%) | 97 (20%) | | 113 (24%) | 110 (23%) | 104 (22%) | 0.57 |
| FPG 100-125 mg/dL, % | 486 (26%) | 120 (25%) | | 114 (24%) | 132 (28%) | 120 (25%) | 0.60 |
| LDL cholesterol, mg/dL | 98 (80, 119) | 101 (81, 122) | | 99 (79, 122) | 96 (81, 116) | 98 (80, 116) | 0.30 |
| eGFR, mL/min/1.73 m^2^ | 119 (106, 131) | 118 (106, 130) | | 117 (106, 128) | 119 (105, 130) | 121 (108, 133) | **0.005** |
| Albuminuria (ACR ≥ 30 mg/g) | 166 (9%) | 31 (6%) | | 34 (7%) | 48 (10%) | 53 (11%) | **0.03** |
| Fibrinogen, mg/dL | 359 (311, 416) | 357 (307, 418) | | 349 (306, 401) | 358 (311, 416) | 372 (320, 429) | **<0.001** |
| PAI-1, ng/mL | 45 (27, 69) | 45 (26, 65) | | 42 (26, 71) | 43 (28, 68) | 48 (29, 73) | 0.14 |
| CRP, mg/L (Visit 4 only) (a) | 3.2 (1.2, 6.9) | 3.1 (1.1, 6.6) | | 2.8 (1.1, 6.0) | 3.4 (1.3, 7.0) | 3.6 (1.5, 8.0) | **0.01** |

SD, standard deviation; IQR, interquartile range; ACR, Albumin to creatinine ratio in urine; HDL, High density lipoprotein; LDL, Low density lipoprotein; eGFR, estimated glomerular function; SBP, systolic blood pressure; DBP, diastolic blood pressure; BMI, body mass index

Values are median (interquartile range) for continuous variables and number of participants (percentage) for categorical variables. P-values from a nonparametric Kruskal-Wallis test of difference in distribution (continuous variables) or Pearson’s chi-square test of independence (categorical variables).

(a) CRP was measured only at Visit 4 in 1,791 participants.
