# Supplementary material for: Low-moderate urine arsenic and biomarkers of thrombosis and inflammation in the Strong Heart Study
Source: PLoS One. 2017 Aug 3;12(8):e0182435. doi: 10.1371/journal.pone.0182435 (PMC5542675; doi:10.1371/journal.pone.0182435)
Supplement: S4 Table — (DOCX) [file pone.0182435.s007.docx]

# S4 Table. Median Concentrations of Plasma Fibrinogen, PAI-1, and CRP in Strong Heart Family Study (SHFS) Participants without Diabetes by Participant Characteristics

|  |  | | | **Visit 3 pilot/Visit 4** | | | | | | **Visit 4** | | |
| --- | --- | --- | --- | --- | --- | --- | --- | --- | --- | --- | --- | --- |
|  |  | | | **Fibrinogen (mg/dL)** | | | | **PAI-1 (ng/mL)** | | **CRP (mg/L)** | | |
|  | **N (%)** | | | **Median** | | **p-value** | | **Median** | **p-value** | **N (%)** | **Median** | **p-value** |
| Age, years (a) |  | | |  | | |  |  |  |  |  |  |
| ≤ 36 | 886 (47%) | | | 345 | | | **<0.001** | 44 | 0.77 | 833 (44%) | 2.3 | **<0.001** |
| > 36 | 1015 (53%) | | | 370 | | |  | 45 |  | 948 (50%) | 3.8 |  |
| Sex |  | | |  | | |  |  |  |  |  |  |
| Male | 758 (40%) | | | 335 | | | **<0.001** | 46 | 0.08 | 697 (37%) | 2.3 | **<0.001** |
| Female | 1143 (60%) | | | 375 | | |  | 43 |  | 1084 (57%) | 4.0 |  |
| Education, % |  | | |  | | |  |  |  |  |  |  |
| < High school | 572 (30%) | | | 356 | | | 0.45 | 42 | 0.48 | 538 (28%) | 2.4 | **<0.001** |
| ≥ High school | 1329 (70%) | | | 361 | | |  | 45 |  | 1243 (65%) | 3.5 |  |
| Smoking |  | | |  | | |  |  |  |  |  |  |
| Never or former | 1177 (62%) | | | 357 | | | 0.10 | 42 | **<0.001** | 1106 (58%) | 3.1 | 0.12 |
| Current | 724 (38%) | | | 363.5 | | |  | 48 |  | 675 (36%) | 3.5 |  |
| Alcohol drinking |  | | |  | | |  |  |  |  |  |  |
| Never or former | 700 (37%) | | | 371 | | | **<0.001** | 40 | **<0.001** | 655 (34%) | 3.2 | 0.34 |
| Current | 1201 (63%) | | | 351 | | |  | 47 |  | 1126 (59%) | 3.2 |  |
| BMI, kg/m^2^ |  | | |  | | |  |  |  |  |  |  |
| < 30 | 976 (51%) | | | 340 | | | **<0.001** | 35 | **<0.001** | 940 (49%) | 1.9 | **<0.001** |
| ≥ 30 | 925 (49%) | | | 384 | | |  | 58 |  | 841 (44%) | 4.8 |  |
| FPG, mg/dL |  | | |  | | |  |  |  |  |  |  |
| < 100 | 1415 (74%) | | | 356 | | | **0.004** | 41 | **<0.001** | 1347 (71%) | 2.9 | **<0.001** |
| ≥ 100 | 486 (26%) | | | 367 | | |  | 55 |  | 434 (23%) | 4.3 |  |
| LDL cholesterol, mg/dL | | |  | |  | |  |  |  |  |  |  |
| <100 | 986 (52%) | | | 359 | | | 0.64 | 42 | **<0.001** | 951 (50%) | 3.1 | **0.01** |
| ≥100 | 915 (48%) | | | 360 | | |  | 47 |  | 830 (44%) | 3.3 |  |
| Hypertension |  | | |  | | |  |  |  |  |  |  |
| No | 1477 (78%) | | | 353 | | | **<0.001** | 42 | **<0.001** | 1380 (73%) | 2.9 | **<0.001** |
| Yes | 424 (22%) | | | 384 | | |  | 51 |  | 401 (21%) | 4.4 |  |
| eGFR, mL/min/1.73 m^2^ | |  | |  | | |  |  |  |  |  |  |
| ≥60 | 1885 (99%) | | | 359 | | | **0.001** | 45 | 0.84 | 1765 (93%) | 3.2 | 0.97 |
| <60 | 16 (1%) | | | 429.5 | | |  | 45 |  | 16 (1%) | 2.9 |  |
| Albuminuria |  | | |  | | |  |  |  |  |  |  |
| No | 1735 (91%) | | | 359 | | | **0.01** | 44 | 0.95 | 1625 (85%) | 3.1 | **0.003** |
| Yes | 166 (9%) | | | 371.5 | | |  | 46 |  | 156 (8%) | 4.0 |  |

eGFR, estimated glomerular function; LDL, low-density lipoprotein; BMI, Body mass index; AZ, Arizona; OK, Oklahoma; ND/SD, North Dakota and South Dakota, FPG, Fasting plasma glucose

(a) Dichotomized at the median value.
