# Supplementary material for: Low-moderate urine arsenic and biomarkers of thrombosis and inflammation in the Strong Heart Study
Source: PLoS One. 2017 Aug 3;12(8):e0182435. doi: 10.1371/journal.pone.0182435 (PMC5542675; doi:10.1371/journal.pone.0182435)
Supplement: S5 Table — (DOCX) [file pone.0182435.s008.docx]

# S5 Table. Adjusted Geometric Mean Ratios (95% Confidence Intervals) of Baseline (Visit 3 pilot/Visit 4) Plasma Fibrinogen, PAI-1, and CRP concentrations in Strong Heart Family Study (SHFS) Participants without Diabetes by Urine Arsenic Concentrations

|  | **Urine Arsenic (Sum iAs, MMA, DMA, µg/g creatinine)** | | | | | | | |
| --- | --- | --- | --- | --- | --- | --- | --- | --- |
|  | **Quartiles** | | | | | **Log-transformed**  **75^th^ vs. 25^th^ percentile (b)** | | **Quadratic**  **Splines (c)** |
|  | **Q1** | | **Q2** | **Q3** | **Q4** |  |  |  |
| **Median:** | **2.3** | | **3.6** | **5.2** | **10.3** | **7.1 vs. 2.9** | **p-value** | **p-value** |
| **Fibrinogen (N=1901)** | | |  |  |  |  |  |  |
|  | |  |  |  |  |  |  |  |
| Model 1 | | 1 (Ref) | 0.99 (0.96, 1.01) | 0.99 (0.96, 1.01) | 1.00 (0.97, 1.03) | 1.00 (0.98, 1.01) | 0.54 | 0.327 |
| Model 2 | | 1 (Ref) | 0.99 (0.96, 1.01) | 0.98 (0.96, 1.01) | 1.00 (0.97, 1.03) | 0.99 (0.98, 1.01) | 0.47 | 0.19 |
| Model 3 (a) | | 1 (Ref) | 0.99 (0.96, 1.01) | 0.98 (0.96, 1.01) | 1.00 (0.97, 1.03) | 0.99 (0.98, 1.01) | 0.31 | 0.118 |
|  | |  |  |  |  |  |  |  |
| **PAI-1 (N=1901)** | | |  |  |  |  |  |  |
|  | |  |  |  |  |  |  |  |
| Model 1 | | 1 (Ref) | 1.01 (0.93, 1.09) | 1.01 (0.93, 1.10) | 1.05 (0.95, 1.15) | 1.01 (0.97, 1.06) | 0.59 | 0.96 |
| Model 2 | | 1 (Ref) | 1.01 (0.93, 1.09) | 1.02 (0.94, 1.11) | 1.05 (0.96, 1.15) | 1.01 (0.97, 1.06) | 0.59 | 0.97 |
| Model 3 (a) | | 1 (Ref) | 1.01 (0.93, 1.09) | 1.02 (0.94, 1.10) | 1.05 (0.96, 1.15) | 1.01 (0.97, 1.06) | 0.57 | 0.93 |
|  | |  |  |  |  |  |  |  |
| **CRP (N=1791) (d)** | | |  |  |  |  |  |  |
|  | |  |  |  |  |  |  |  |
| Model 1 | | 1 (Ref) | 0.88 (0.77, 1.01) | 0.96 (0.83, 1.10) | 0.93 (0.80, 1.09) | 0.96 (0.89, 1.04) | 0.30 | **<0.001** |
| Model 2 | | 1 (Ref) | 0.88 (0.76, 1.01) | 0.94 (0.82, 1.09) | 0.91 (0.77, 1.07) | 0.95 (0.87, 1.02) | 0.16 | **<0.001** |
| Model 3 (a) | | 1 (Ref) | 0.88 (0.76, 1.01) | 0.94 (0.81, 1.08) | 0.89 (0.76, 1.05) | 0.94 (0.86, 1.01) | 0.10 | **<0.001** |
|  | |  |  |  |  |  |  |  |

Model 1 adjusted for age, sex, and education (no, some, or finished high school), smoking (never, former, current), and alcohol drinking (never, former, current), BMI (kg/m^2^), LDL cholesterol (mg/dL), hypertension (yes/no), and eGFR (mL/min/1.73 m^2^).

Model 2 was further adjusted for study center (Arizona, Oklahoma, North and South Dakota).

Model 3 was further adjusted for albuminuria (ACR <30 mg/g, >30 to <300 mg/g, and ≥300 mg/g) and fasting glucose (mg/dL).

(a) Fasting glucose was available in 99.8% of participants.

(b) Geometric mean ratio comparing the 75^th^ percentile to the 25^th^ percentile of urine arsenic (2.9 and 7.1 µg/g creatinine, respectively). Coefficient of log-transformed arsenic concentrations multiplied by the ratio between the 75^th^ and 25^th^ percentiles.

(c) P-value from a Wald test that the two non-linear restricted quadratic spline coefficients are different from zero. Restricted quadratic splines were created from log-transformed arsenic concentrations by diabetes status, with knots at the 10^th^, 50^th^, and 90^th^ percentiles.

(d) CRP was only measured at Visit 4.
