# Supplementary material for: Low-moderate urine arsenic and biomarkers of thrombosis and inflammation in the Strong Heart Study
Source: PLoS One. 2017 Aug 3;12(8):e0182435. doi: 10.1371/journal.pone.0182435 (PMC5542675; doi:10.1371/journal.pone.0182435)
Supplement: S6 Table — (DOCX) [file pone.0182435.s009.docx]

# S6 Table. Geometric Mean Ratios (95% Confidence Intervals) for Baseline Fibrinogen, Visit PAI-1, and Visit 2 CRP in Relation to Baseline Urine Arsenic in SHFS Participants Without Diabetes by Baseline Participant Characteristics

|  | **Fibrinogen** | | | **PAI-1** | | | **CRP** | | |
| --- | --- | --- | --- | --- | --- | --- | --- | --- | --- |
| **Characteristic** | **N** | **GMR (95% CI)** | **p-interaction** | **N** | **GMR (95% CI)** | **p-interaction** | **N** | **GMR (95% CI)** | **p-interaction** |
| Age |  |  |  |  |  |  |  |  |  |
| <55 y | 886 | 1.02 (1.00, 1.04) |  | 886 | 1.01 (0.95, 1.08) |  | 833 | 1.01 (0.91, 1.13) |  |
| ≥55 y | 1015 | 1.00 (0.98, 1.02) | 0.10 | 1015 | 1.01 (0.96, 1.06) | 0.95 | 948 | 0.97 (0.88, 1.07) | 0.54 |
| Sex |  |  |  |  |  |  |  |  |  |
| Men | 758 | 1.01 (0.99, 1.03) |  | 758 | 1.00 (0.94, 1.07) |  | 697 | 1.03 (0.92, 1.16) |  |
| Women | 1143 | 1.01 (0.99, 1.02) | 0.62 | 1143 | 1.01 (0.96, 1.06) | 0.87 | 1084 | 0.94 (0.86, 1.02) | 0.17 |
| Education |  |  |  |  |  |  |  |  |  |
| <High school | 572 | 1.02 (1.00, 1.05) |  | 572 | 1.00 (0.93, 1.07) |  | 538 | 1.00 (0.88, 1.13) |  |
| ≥High school | 1329 | 1.00 (0.99, 1.02) | 0.12 | 1329 | 1.01 (0.96, 1.06) | 0.78 | 1243 | 0.97 (0.89, 1.05) | 0.67 |
| BMI |  |  |  |  |  |  |  |  |  |
| <30 kg/m^2^ | 976 | 1.01 (0.99, 1.03) |  | 976 | 1.01 (0.95, 1.07) |  | 940 | 0.97 (0.88, 1.07) |  |
| ≥30 kg/m^2^ | 925 | 1.01 (0.99, 1.03) | 0.82 | 925 | 1.02 (0.96, 1.09) | 0.72 | 841 | 0.97 (0.87, 1.08) | 0.94 |
| LDL Cholesterol |  |  |  |  |  |  |  |  |  |
| <100 mg/dL | 986 | 0.99 (0.97, 1.01) |  | 986 | 1.02 (0.97, 1.08) |  | 951 | 0.92 (0.84, 1.02) |  |
| ≥100 mg/dL | 915 | 1.02 (1.00, 1.04) | **0.02** | 915 | 0.99 (0.93, 1.05) | 0.37 | 830 | 1.02 (0.92, 1.12) | 0.15 |
| Hypertension |  |  |  |  |  |  |  |  |  |
| No | 1477 | 1.01 (0.99, 1.02) |  | 1477 | 1.00 (0.96, 1.05) |  | 1380 | 0.95 (0.88, 1.03) |  |
| Yes | 424 | 1.01 (0.98, 1.03) | 0.87 | 424 | 1.03 (0.95, 1.11) | 0.58 | 401 | 1.03 (0.90, 1.18) | 0.32 |
| eGFR |  |  |  |  |  |  |  |  |  |
| >60 ml/min/1.73m^2^ | 1885 | 1.01 (0.99, 1.02) |  | 1885 | 1.02 (0.97, 1.06) |  | 1765 | 0.97 (0.91, 1.05) |  |
| ≤60 ml/min/1.73m^2^ | 16 | 0.96 (0.81, 1.14) | 0.59 | 16 | 1.08 (0.64, 1.81) | 0.83 | 16 | 0.55 (0.23, 1.31) | 0.20 |
| Smoking |  |  |  |  |  |  |  |  |  |
| Never | 791 | 1.01 (0.99, 1.02) |  | 791 | 1.02 (0.97, 1.06) |  | 737 | 0.97 (0.91, 1.05) |  |
| Former | 386 | 1.01 (0.99, 1.03) |  | 386 | 0.98 (0.92, 1.04) |  | 369 | 0.98 (0.88, 1.10) |  |
| Current | 724 | 1.01 (0.99, 1.03) | 0.64 | 724 | 0.98 (0.92, 1.04) | 0.45 | 675 | 0.98 (0.88, 1.10) | 0.27 |
| Alcohol Use |  |  |  |  |  |  |  |  |  |
| Never | 214 | 1.01 (0.99, 1.02) |  | 214 | 1.02 (0.97, 1.06) |  | 198 | 0.97 (0.91, 1.05) |  |
| Former | 486 | 1.03 (0.99, 1.07) |  | 486 | 0.97 (0.86, 1.09) |  | 457 | 0.95 (0.77, 1.18) |  |
| Current | 1201 | 1.03 (0.99, 1.07) | 0.24 | 1201 | 0.97 (0.86, 1.09) | 0.19 | 1126 | 0.95 (0.77, 1.18) | 0.37 |
|  |  |  |  |  |  |  |  |  |  |
